# Exosomes derived from HTLV-1 infected cells contain viral proteins and mRNA

**DOI:** 10.1186/1742-4690-12-S1-P98

**Published:** 2015-08-28

**Authors:** Elizabeth Jaworski, Rachel Van Duyne, Sergey Iordanskiy, Philippe Afonso, Gavin Sampey, Myung Chung, Anastas Popratiloff, Renaud Mahieux, Fatah Kashanchi

**Affiliations:** 1 George Mason University, School of Systems Biology, Laboratory of Molecular Virology, Manassas, VA 20110, USA; 2 Center for Cancer Research, National Cancer Institute, National Institutes of Health, Frederick, MD 21702, USA; 3 The George Washington University, Center for Microscopy and Image Analysis, Washington, DC 20037, USA; 4 Equipe Oncogenese Retrovirale, Equipe labelisee 'Ligue Nationale Contre le Cancer', International Center for Research in Infectiology, INSERM U1111 - CNRS UMR5308, Ecole Normale SupÃ©rieure de Lyon, UniversitÃ© Lyon 1, Lyon, 69364 Cedex 07, France

## 

The HTLV-1 transactivator protein Tax controls many critical cellular pathways including host cell DNA damage response mechanisms, cell cycle progression, and apoptosis. Recently, exosomes have been shown to play critical roles during pathogenic viral infections as delivery vehicles for host and viral components including proteins, mRNA and miRNA. We hypothesized that exosomes derived from HTLV-1 infected cells contain unique host and viral proteins that may contribute to pathogenesis. We have characterized exosomes released from uninfected and HTLV-1 infected cell lines, as well as ATL and HAM/TSP material. The functional impact of exosomes derived from HTLV-1 infected cells on naive recipient cells was evaluated by utilizing transcription and reactive oxygen species (ROS) assays. Exosomes from HTLV-1 infected cells displayed unique proteomic profiles distinct from exosomes derived from uninfected cells. For instance, proinflammatory mediators are contained within the exosomes, as well as viral mRNA transcripts including Tax, HBZ, and Env. We found that exosomes from infected cells deliver functional Tax to naÃ¯ve recipient cells as well as cytokines. The release of factors was through Calcium and Calcium channels. We observed that exosomes released from HTLV-1 infected, Tax-expressing cells contributed to enhanced survival of target cells treated with α-FAS. Two other critical proteins were found in these exosomes that could contribute to overall activation of neighboring cells including Tax1BP1 and PrP. Both proteins are critical for activation of NFkB patway in recipient cells. Collectively, our results suggest that exosomes may play an important role in extracellular delivery of functional HTLV-1 proteins and mRNA to recipient cells. Furthermore, exosomes derived from infected cells are capable of inducing an ROS response in naÃ¯ve cells and contribute to an anti-apoptotic phenotype in cells treated with α-FAS.

